# Clinical Implications of “Atypia” on Biopsy: Possible Precursor to Lung Cancer?

**DOI:** 10.3390/curroncol28040228

**Published:** 2021-07-06

**Authors:** Denise Albano, Lee Ann Santore, Thomas Bilfinger, Melissa Feraca, Samantha Novotny, Barbara Nemesure

**Affiliations:** 1 Lung Cancer Evaluation Center at Stony Brook University Hospital, Stony Brook, NY 11790, USA; Thomas.Bilfinger@stonybrookmedicine.edu (T.B.); melissa.feraca@stonybrookmedicine.edu (M.F.); barbara.nemesure@stonybrookmedicine.edu (B.N.); 2 Renaissance School of Medicine at Stony Brook University, Stony Brook, NY 11790, USA; lee.santore@stonybrookmedicine.edu (L.A.S.); samantha.novotny@stonybrookmedicine.edu (S.N.)

**Keywords:** atypia, lung cancer, lung nodule, PET, cytology

## Abstract

Background: It is common for biopsies of concerning pulmonary nodules to result in cytologic “atypia” on biopsy, which may represent a benign response or a false negative finding. This investigation evaluated time to diagnosis and factors which may predict an ultimate diagnosis of lung cancer in these patients with atypia cytology on lung nodule biopsy. Methods: This retrospective study included patients of the Stony Brook Lung Cancer Evaluation Center who had a biopsy baseline diagnosis of atypia between 2010 and 2020 and were either diagnosed with cancer or remained disease free by the end of the observation period. Cox Proportional Hazard (CPH) Models were used to assess factor effects on outcomes. Results: Among 106 patients with an initial diagnosis of atypia, 80 (75%) were diagnosed with lung cancer. Of those, over three-quarters were diagnosed within 6 months. The CPH models indicated that PET positivity (SUV ≥ 2.5) (HR = 1.74 (1.03, 2.94)), nodule size > 3.5 cm (HR = 2.83, 95% CI (1.47, 5.45)) and the presence of mixed ground glass opacities (HR = 2.15 (1.05, 4.43)) significantly increased risk of lung cancer. Conclusion: Given the high conversion rate to cancer within 6 months, at least tight monitoring, if not repeat biopsy may be warranted during this time period for patients diagnosed with atypia.

## 1. Introduction

Lung cancer incidence and mortality rates continue to rank among the highest of all cancer types in the United States [1]. Pulmonary nodule size and growth rate are known risk factors for lung cancer, [2] with nodules ≥ 8 mm posing a higher likelihood of malignancy [3]. Concerning scans typically warrant tissue sampling to assist with initial diagnosis [4]. The most accessible sites are usually chosen for biopsy, [5] as different approaches carry different yields [6]. As it is not uncommon for tissue samples to be classified as having atypical cellularity, repeat biopsies or surgical interventions are further recommended [7]. It remains unclear, however, how often a cytologic diagnosis of atypia is ultimately found to be a malignancy, and in what time frame this lung cancer diagnosis occurs. The purpose of this study was to evaluate the duration of time and potential risk factors associated with the development of lung cancer among patients with an initial diagnosis of atypia.

## 2. Materials and Methods

This study retrospectively examined the electronic medical records (EMR) of all patients seen by the Lung Cancer Evaluation Center (LCEC) at the Stony Brook Cancer Center, Stony Brook, NY, USA, between 1 January 2010 and 31 May 2020. The LCEC was established to evaluate, diagnose, treat, and monitor patients found to have concerning pulmonary nodules. The LCEC maintains a database which captures longitudinal data for each patient, beginning with their baseline visit. The surveillance component of the program enables an opportunity to evaluate patients who span the full spectrum of disease and includes those who are diagnosed with cancer at first presentation, those who present with benign nodules and develop cancer over time, and those who present with non-malignant lesions yet remain cancer-free while under care of the program.

Upon identification of a suspicious nodule on imaging, a PET is ordered for further evaluation. If found to be FDG avid, a biopsy is recommended. A biopsy that yields atypia is presented at the weekly tumor board. Upon consensus of the multidisciplinary care team, which includes representation from all program sub-specialties, an individualized patient plan is formulated. In most cases, if the images are suspicious and biopsy results are atypical, the team will recommend a repeat scan and possible repeat procedure within 1–6 months [8].

Data for this investigation, which were abstracted from both the LCEC database and EMR, included age, gender, atypia status, date of atypia diagnosis, smoking history (current, former, or never), comorbidities (history of diabetes, hypertension, and emphysema), family history of cancer, and nodule features including lesion size (in cm), mixed ground glass opacity type, spiculation, location (upper vs. lower lobe, right vs. left) and PET SUV. PET positivity was defined as PET SUV ≥ 2.5 [9,10]. Additionally, for those patients who were ultimately diagnosed with cancer, the date of cancer diagnosis was collected. For patients who remained disease-free during the observation period, the last date of examination was recorded.

**Figure 1:**
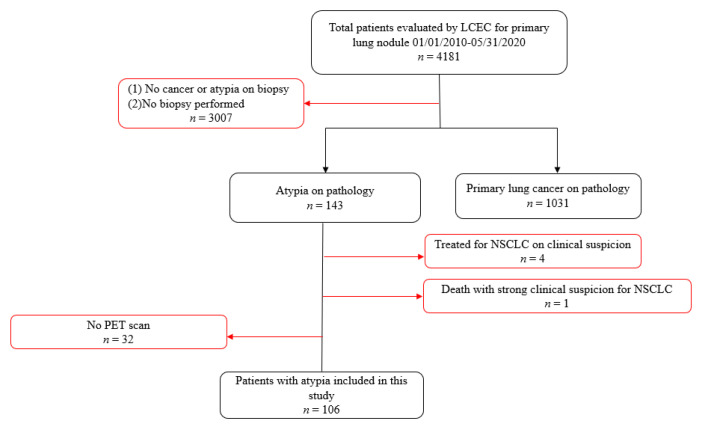
Inclusion and exclusion criteria for this study.

Figure 1 presents the inclusion and exclusion criteria for the study sample. As this study sought to evaluate cytology-proven outcomes among patients with atypia, those who did not have a lung biopsy or a diagnosis of atypia (*n* = 3007) were excluded from the study, as were patients found to have biopsy-confirmed primary lung cancer without a previous diagnosis of atypia (*n* = 1031). Among the 143 patients receiving a baseline diagnosis of atypia, 4 were treated as a result of a strong clinical suspicion of lung cancer despite the biopsy confirmation of only atypia without re-biopsy, and 1 died prior to such confirmation. Those patients were excluded from the study, along with 32 others who did not have a PET scan. The remaining 106 patients represent the basis for this investigation.

### Statistical Analyses

Descriptive statistics were used to compare demographic and nodule characteristics between patients with atypia who were ultimately diagnosed with lung cancer and those who remained disease-free by the conclusion of the study. Quantitative variables are presented as mean ± standard deviation (SD), and differences between groups are evaluated using the Student’s *t*-test. Categorical factors are presented as percentages and compared using the chi-square test. The distribution of duration in time (in days) between the date of atypia diagnosis and the date of final outcome (defined as either diagnosis of lung cancer or disease-free at the close of the study) is presented in quartiles. Kaplan–Meier curves are provided, and patients for whom no event occurred within the specified time frame were right censored. Cox Proportional Hazard (CPH) Models, adjusted for age and gender, were used to assess the possible effect of any variable on outcome status. Factors found to be different between groups at the 0.10 level of significance in the univariate analyses were included in the CPH Models. Hazard Ratios (HR) and 95% Confidence Intervals (CI) are presented. SPSS version 21 (IBM Corporation, Armonk, NY, USA) was used to carry out the analyses for this study.

## 3. Results

Among 106 patients with a baseline diagnosis of atypia, 80 (75%) were diagnosed with lung cancer during the observation period. While adenocarcinoma (63%) and squamous cell (15%) were the primary cancer types subsequently diagnosed among cases, pneumonia (23%), fibrosis (19%), and chronic inflammation (12%) were the primary histologic diagnoses among patients who remained cancer-free.

Table 1 presents the demographic and nodule characteristics of the study sample, stratified by cancer outcome status. The average age of lung cancer among cases was 67.4 years and 47.5% were male. There were no significant differences in age or gender between patients who were diagnosed with cancer and those who remained disease-free by the close of the study. Likewise, there were no differences in smoking status, history of diabetes or hypertension, family history of cancer, or diagnosis of emphysema between the two groups. Cancer cases were more likely to be PET positive (*p* = 0.02) and had marginally larger baseline nodules, on average, than patients without cancer (2.4 cm vs. 1.9 cm, *p* = 0.08). Additionally, mixed ground glass opacities tended to be more common among patients diagnosed with lung cancer compared to non- cancer cases (*p* = 0.06). As solid and mixed ground glass nodules represent different entities of disease, patients were further stratified by opacity type to determine if there were any differences between the two groups. Cases with mixed ground glass opacities were found to be older than those with solid nodules (73.6 ± 6.0 vs. 66.5 ± 8.9 years, *p* = 0.02), however there were no other statistically significant differences with regard to any of the demographic factors or nodule characteristics under investigation.

**Figure 2:**
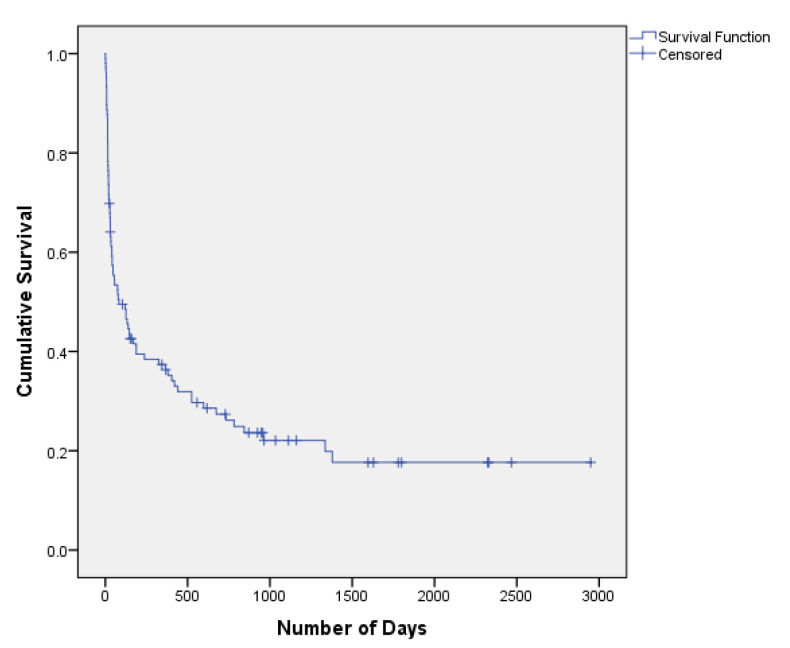
Kaplan–Meier Survival Curve for Patients Diagnosed with Atypia.

The distribution of duration in days between the baseline diagnosis of atypia and the final outcome is presented in Table 2, alongside the Kaplan–Meier survival curve (Figure 2). Among lung cancer cases, the median amount of time to diagnosis after an initial indication of atypia was 35 days, and more than 75% of patients were diagnosed within 6 months. Less than 20% of cases were diagnosed after 1 year. In contrast, 75% of non-cases remained disease-free after 1 year. Additionally, similar conversion rates were found for patients with solid (75%) and mixed ground glass lesions (70%) at 6 months.

CPH Models were used to determine factors that may be predictive of lung cancer among patients with atypia. The results are presented in Table 3 and indicate that PET positivity (HR = 1.74 (1.03, 2.94)), nodule size > 3.5 cm (HR = 2.83 (1.47, 5.45)), and mixed ground glass opacity type (HR = 2.15 (1.05, 4.43)) were significant predictors of cancer.

As more than 80% of cases were diagnosed within 12 months and 25% of non-cases were not under the care of the LCEC for a full year during the study period, additional CPH models were performed to address any potential misclassification biases. After excluding non-cases who did not participate in the program for a minimum of 365 days, the main findings remained unchanged.

## 4. Discussion

While the literature indicates that larger and more rapidly growing pulmonary nodules have an increased likelihood of carcinogenesis [2] and that nodules > 8 mm in size pose a higher likelihood of malignancy, [3] data are more limited as to whether (and in what capacity) atypical cells may represent precursors to cancer. Findings from the current investigation indicate that 75% of patients with an initial diagnosis of atypia were diagnosed with cancer. Of those, half were diagnosed within one month, and more than three-quarters were confirmed to have lung cancer within 6 months. Additionally, PET positive patients with mixed ground glass opacities > 3.5 cm in size were at significantly increased risk of developing lung cancer. The findings from this study suggest that patients with this profile who are found to have a result of “Atypia” on their biopsy may be considered as likely already having cancer, and this may warrant repeat attempts at establishing a definitive diagnosis to potentially reduce treatment delays.

Many factors may contribute to a diagnosis of atypical cellularity [11,12] and the outcome is often the result of a limited number of cells captured for evaluation. Challenges in obtaining an adequate sample may result from a small or less than desirable location for the biopsy, patient body habitus, expertise of the proceduralist, and the number of times a procedure is performed. Additionally, multiple passes are known to reduce the quality and quantity of the specimens [2,13]. Sampling techniques, including fine needle aspiration, CT or ultrasound guided percutaneous procedures, and bronchoscopy [4] can also play a role in the adequacy of the sample obtained. CT-guided transthoracic lung biopsies have been reported to produce a 77–90% diagnostic yield, [14,15] whereas bronchoscopy has been shown to have a 70–81% yield, and bronchial brushes a 56% yield [16,17]. The procedure type, as well as the size and location of the nodule, remain the main factors influencing sample collection. Lesions larger than 1 cm and at a location less than 4 cm from the needle path are thought to provide the most significant yield [18].

Progression of benign nodules to cancer is often quantified in terms of doubling time. The literature indicates that cancerous nodules are typically expected to double in volume between 20 and 300 days [19,20,21,22]. This study found that 50% of patients with atypia who were diagnosed with lung cancer had biopsy-proven cancer within one month, and 75% within 6 months. The short interval of time between original atypia cytology and final cancer diagnosis noted in this investigation may be attributable, at least in part, to the multidisciplinary approach employed by the LCEC program. In most cases, if images are suspicious and the cell type is atypical, a repeat scan and possible repeat procedure will be recommended within 1–6 months. This tight surveillance practice may potentially reduce the time to diagnosis among this subgroup of patients.

This investigation is among the first to evaluate factors influencing the ultimate diagnosis of lung cancer after findings of atypical cytology in a lung nodule biopsy specimen, however it has several limitations. First, given its observational, retrospective design, this study lacked data for several important factors that may influence outcomes. Details were unavailable for biopsy yield and adequacy, as well as the molecular and cellular profiles of the sample. Without this information, it is difficult to evaluate the mechanistic drivers which may have influenced the cytologic determination of atypia. Secondly, the study was unable to capture and quantify variables such as the expertise of the proceduralist, the physical environment, transport conditions of the sample to the cytology lab, and the experience of the pathologist, all of which may have contributed to the resulting diagnosis of atypia. In this investigation, 98% of the biopsies were performed at Stony Brook and reviewed by experienced pathologists specializing in the lung. While the experience of the pathologists external to the Stony Brook Cancer Center is unknown and may introduce potential bias, the direction of such bias is unclear, and the low percentage of patients with an external review suggests that such bias would be minimal. Lastly, the study’s sample size was limited and the investigation was conducted within a single institution in the northeast, thereby potentially limiting the generalizability of the findings. Future studies are needed to validate the results of this investigation and further elucidate the mechanisms and factors that may influence the conversion of atypical cells to cancer.

## 5. Conclusions

In patients with lung nodules initially classified as atypia on biopsy, repeat efforts at establishing a diagnosis are warranted in order to cut treatment delays to a minimum due to the high likelihood of this population already having a cancer; particularly within the first 6 months of the baseline biopsy. As such, further consideration should be given to repeat biopsy. Patients with mixed ground glass pulmonary nodules > 3.5 cm in size and a positive PET scan may particularly benefit from tighter monitoring in the early months following a diagnosis of atypia. Targeting this subgroup of patients may help to maximize the potential for early detection and thereby improve patient outcomes.

## Figures and Tables

**Table 1:** Demographic and Pulmonary Nodule Characteristics of N = 106 Patients Diagnosed with Atypia Between January 2010 and May 2020 at the Stony Brook Lung Cancer Evaluation Center, Stratified by Lung Cancer Status.

Characteristic	Lung Cancer (*n* = 80)	No Cancer (*n* = 26)	*p*-Value
Demographic Factors			
Age at diagnosis, year	67.4 ± 9.3	64.9 ± 11.7	0.27
Gender, % male	47.5	61.5	0.21
Smoking History, %			0.28
Never	6.5	16.7	
Former	64.9	62.5	
Current	28.6	20.8	
History of Diabetes, %	31.5	16.0	0.13
History of Hypertension, %	61.1	68.0	0.54
Family History of Cancer, %	38.2	44.0	0.62
History of Emphysema, %	9.7	4.0	0.37
Nodule Features			
PET SUV ≥ 2.5,%	73.8	50.0	0.02
Size of Nodule at Baseline (cm)	2.4 ± 1.5	1.9 ± 0.9	0.08
Mixed Ground Glass Opacity, %	12.5	0.0	0.06
Spiculated, %	16.3	15.4	0.92
Location of Nodule			
LLL	8.8	7.7	0.19
LUL	26.3	23.1	
Lymph	6.3	0.0	
RLL	22.5	26.9	
RML	1.3	11.5	
RUL	35.0	30.8	

Note: LLL = left lower lobe. LUL = left upper lobe. RLL = right lower lobe. RML = right middle lobe. RUL = right upper lobe.

**Table 2:** Quartile Distribution of the Number of Days from Initial Diagnosis of Atypia to Final Outcome, Stratified by Disease Status.

Quartile Percentage	Number of Days from Atypia Diagnosis to Final Outcome	
	Lung Cancer	No Cancer
25th	15	361
50th	35	953
75th	165	1667

**Table 3:** Cox Proportional Hazard Model for Patients with a Baseline Diagnosis of Atypia.

Characteristic	HR (95% CI)	*p*-Value
Baseline Age (years)	1.00 (0.98, 1.02)	0.92
Male Gender	1.12 (0.71, 1.73)	0.65
PET Positive *	1.74 (1.03, 2.94)	0.04
Size of Nodule (>3.5 cm)	2.83 (1.47, 5.45)	<0.01
Mixed Ground Glass Opacity	2.15 (1.05, 4.43)	0.04

* PET SUV ≥ 2.5.

## Data Availability

The data presented in this study are available on request from the corresponding author. The data are not publicly available due to patient privacy.
